# Psychological contribution to understanding the nature of dry eye disease: a cross-sectional study of anxiety sensitivity and dry eyes

**DOI:** 10.1080/21642850.2020.1770093

**Published:** 2020-05-28

**Authors:** Marko Toth, Nataša Jokić-Begić

**Affiliations:** a Department of Optometry, University of Applied Sciences Velika Gorica, Velika Gorica, Croatia; b Department of Psychology, Faculty of Humanities and Social Sciences, University of Zagreb, Zagreb, Croatia

**Keywords:** Anxiety sensitivity, dry eye disease (DED), depression, anxiety, quality of life

## Abstract

Dry eye disease (DED) represents a common health problem in the general population. Previous studies have demonstrated that the subjective symptoms of dry eye are associated with several psychological factors, including depression, anxiety and post-traumatic stress disorder. However, there is a lack of empirical information about the mechanisms underlying the relationships between DED and various psychological symptoms. In light of emerging evidence of its trans-diagnostic nature, anxiety sensitivity (i.e. AS) represents one promising factor for further understanding DED. The present study aimed to explore whether anxiety sensitivity plays a role in the perception of DED symptoms in a community-based sample of adults aged 20–89 years (*N* = 381; *M* = 39.72, *SD* = 12.6). A dry ocular surface was reported by 22.8% of the participants. As expected, women more often reported symptoms of dry eye that could be categorized as moderate to severe. The findings demonstrated that AS, and the AS-psychological concerns dimension in particular, predict the intensity of dry eye symptoms above and beyond depressive and anxiety symptoms. These findings add to a growing body of work underscoring the relevance of AS in increasing the risk of chronic medical conditions.

## Introduction

### Background/rationale

With a prevalence reaching up to one third of the general population, dry eye disease (DED) is a growing health problem (Gayton, 2009). In a recent systematic review of the literature on the economic and humanistic impacts of dry eye worldwide, McDonald, Patel, Keith, and Snedecor (2016) concluded that the indirect costs of DED represent the substantial proportion of the overall cost of lost work productivity productivity in the United States, Western Europe and Japan. In addition to its large negative impact on physical function and health-related quality of life, DED also has a potential impact on psychological function. Furthermore, only 12% of people with normal eyes (i.e. without DED) do not experience any symptoms of DED whatsoever (Shimmura, Shimazaki, & Tsubota, 1999). Together, these facts confirm that DED represents a problem that widely affects public health.

Dry eye disease is characterized by symptoms of eye dryness, irritation and a gritty sensation of foreign particles in the eye, accompanied by light sensitivity and an itching sensation (Stevenson, Chauhan, & Dana, 2012). Most research indicates that discomfort and dryness are the most frequent and intense symptoms. In a study examining DED in a large Japanese sample Shimmura et al. (1999) found that eye fatigue is the most frequent symptom in both people with dry eyes (80%) and people with normal eyes (47%). These symptoms worsened over the course of the day and were reported to be quite bothersome (Begley, Caffery, Chalmers, & Mitchell, 2002; Begley, Caffery, Nichols, & Chalmers, 2000; Begley, Chalmers, Albetz et al., 2003; Begley, Chalmers, Mitchell et al., 2001). In our review of 14 different questionnaires that partially examine the symptoms of dry eye (as part of larger epidemiological studies) or are specifically designed for dry eye diagnostic purposes confirmed that the most commonly included symptom in questionnaires is eye dryness (86%), followed by eye pain or ache (64%) and a stinging or burning sensation (64%) (Toth, 2018).

A number of common activities of daily life, such as reading and driving, are adversely affected by this condition (Miljanović, Dana, Sullivan, & Schaumberg, 2007) because of its association with reduced quality of life, as it relates to visual function (Mizuno, Yamada, Myake, & Dry Eye Survey Group of the National Hospital Organization of Japan, 2010) and general health (Asiedu, Dzasimatu, & Kyei, 2018; Friedman, 2010; Miljanović et al., 2007; Paulsen et al., 2014; Rossi, Tinelli, Pasinetti, Milano, & Bianchi, 2009).

There are a number of risk factors for DED including: female gender, advancing age, windy, smoky, or dry environments, exposure to dust or sun (Khurana, Choudhary, Ahluwalia, & Gupta, 1991), prolonged periods of screen time, autoimmune disease and eye surgery, contact lens use, previous treatment for dry eye, medication, unusual eye sensitivity, mucous membrane dryness and waking irritation (Tan, Morgan, Cai, & Straughan, 2015). Many studies have found little or no correlation between the severity of symptoms and clinical diagnostic test results (Baudouin et al., 2014; Begley et al., 2003; Kawashima et al., 2015; Lavrič & Olup, 2010; Mizuno et al., 2010; K.K. Nichols, Nichols, & Mitchell, 2004; Pult, Purslow, & Murphy, 2011; Schein, Tielsch, Muñoz, Bandeen-Roche, & West, 1997; Schiffman, Christianson, Jacobsen, Hirsch, & Reis, 2000). Furthermore, the symptoms of DED have been found to be more aligned to non-ocular conditions than to tear film parameters (Galor et al., 2015). DED has recently been associated with other chronic pain (Shtein et al., 2016; Stapleton et al., 2017; Vehof, Smitt-Kamminga, Kozareva, Nibourg, & Hammond, 2016), anxiety and depression (Stapleton et al., 2017; Wan, Chen, & Young, 2016; Wen et al., 2012; Yilmaz, Gokler, & Unsal, 2015) and somatoform disorders (Stapleton et al., 2017).

In recent years, there has been accumulating evidence that psychological characteristics such as neuroticism, anxiety and depression play a leading role in the perception of dry eye symptoms (Ichinohe et al., 2016; Kawashima et al., 2015; Kim et al., 2011; Mizuno et al., 2010; Um et al., 2018; Wen et al., 2012). A recent meta-analysis demonstrated that the prevalence of depression and anxiety is approximately three times higher among patients with DED, irrespective of the underlying etiology of DED and patient ethnicity (Wan et al., 2016). DED has also been shown to be associated with suicidal ideation, even after controlling for depression level (Um et al., 2018). Most recently, DED was the only common ocular disorder found to be associated with an increased depression score (Jonas et al., 2018) and showed to be closely related to psychological stress (Hyon, Yang, & Han, 2019).

Although the growing literature confirms the relationship between DED and psychological variables, none of them, to our knowledge, explored the relation between DED and anxiety sensitivity (AS). We propose that anxiety sensitivity (AS) might play an important role in explaining this observed relationship between dry eye disease and various psychological syndromes. AS as a construct was first proposed by Reiss and McNally (1985). It refers to a person's tendency to fear anxiety-related symptoms (e.g. racing heart, blushing, breathing problems) due to the belief that there will be some harmful physical, social or mental consequences as a result of having these symptoms. Although originally conceived AS as a unitary construct (Reiss & McNally, 1985) it has proven to be a hierarchical and multidimensional and consisted of three lower-order factors (Physical Concerns, Psychological Concerns, and Social Concerns) and a single higher-order factor (global AS) (Zinbarg, Mohlman, & Hong, 1999). AS is related to a variety of anxiety disorders (particularly panic disorder) and non-anxiety psychopathology like depression, hypochondria, addictions and chronic pain disorders (Cox, Borger, & Enns, 1999; Deacon & Abramowitz, 2006; Reiss, 1987, 1991). Recent studies suggest that AS might reflect a broader tendency to fear somatic symptoms more generally, rather than anxiety symptoms specifically (Horenstein, Potter, & Heimberg, 2018). AS is distinct from trait anxiety (Reiss, 1997) and has been found to predict various anxious responses to challenge and stress beyond trait anxiety. Extensive research supports the characterization of AS as a trans-diagnostic vulnerability factor contributing to the development, exacerbation and maintenance of psychological and chronic health conditions (Avallone, McLeish, Luberto, & Bernstein, 2012; Bravo & Silverman, 2001). In this growing area of research, numerous studies have demonstrated a relationship between AS and chronic pain conditions (e.g. fibromyalgia, arthritis and migraines), cardiovascular disease (Norman & Lang, 2005; Seldenrijk et al., 2013), gastrointestinal symptoms, asthma, vestibular dysfunction and tinnitus (Avallone et al., 2012; Hazlett-Stevens, Craske, Mayer, Chang, & Naliboff, 2003; Smitherman, Davis, Walters, Young, & Houle, 2015), diabetes (Anderbro et al., 2015), psoriasis (Dixon et al., 2016), and post-concussion syndrome (e.g. Broshek, De Marco, & Freeman, 2015).

One potential explanation for the relationship between AS and various chronic health conditions lies in the hypothesis that AS represents broader beliefs about the harmfulness not only of anxiety symptoms, but rather of all interoceptive sensations. Individuals with high levels of anxiety sensitivity are prone to be hypervigilant to both pain and other bodily sensations and to catastrophically misinterpret these sensations as more threatening and dangerous. In light of the fact that the main symptoms of dry eye disease are discomfort, dryness, irritability, pain and fatigue, it seems reasonable to assume that AS plays a role in the perception of dry eyes. To date, however, AS has not been examined in relation to the symptoms of DED.

### Objectives

The present study aimed to explore whether anxiety sensitivity plays a role in the perception of DED symptoms. Specifically, we investigated the robustness of this association by examining the link between DED and AS and a number of AS sub-factors, after controlling for depression and anxiety symptoms.

## Methods

### Study design

An online cross-sectional study was conducted on a convenient community-based sample of adults aged 20–89 in order to explore whether anxiety sensitivity plays a role in the perception of DED. This study used a survey constructed in the online SurveyMonkey® application.

### Setting

The online survey was distributed in Croatia during the month of February 2018. The first point of questionnaire dissemination was via social networks. In order to reach individuals from older age groups, who typically use social networks less than young people, the online questionnaire was also disseminated via personal mailing lists. Finally, in an effort to achieve a greater level of sample heterogeneity, further recruitment of participants was based on a snowball technique where diversified social networks, mailing lists and individuals were used as starting points. The anonymous and voluntary nature of participation including the right to be informed was clearly stated and emphasized in the beginning of the survey and survey invitation and participants were able to withdraw from participation at any time. The initiation of questionnaire completion was stated as the respondent’s consent to participation, which was clearly stated in the initial instructions provided to respondents. The research design was approved by the Psychology department's Ethical board in the Faculty of humanities and Social Sciences Zagreb.

### Participants

Any person of legal age living in Croatia and willing to participate voluntarily were eligible for the study. The survey was distributed via personal contacts (e-mail), social networks and a webpage. The first point of questionnaire dissemination was via social networks. In order to reach individuals from older age groups, who typically use social networks less than young people, the online questionnaire was also disseminated via personal mailing lists. Finally, in an effort to achieve a greater level of sample heterogeneity, further recruitment of participants was based on a snowball technique where diversified social networks, mailing lists and individuals were used as starting points.

### Variables

The investigated predictors in the first hierarchical multiple regression were gender, age, Depression, Anxiety and Stress Scale (DASS-21) total score and Anxiety Sensitivity Index (ASI) total score.

The investigated predictors in the second hierarchical multiple regression were gender, age, ASI subscales: physical concerns, psychological concerns and social concerns, and DASS-21 subscales: depression, anxiety and stress.

The outcome variable in both hierarchical multiple regressions was Ocular surface Disease Index total score.

### Data sources/measurement

*Demographic information* included age, gender, educational level, and working status.

*Inclusion criteria information* obtained questions about wearing contact lenses, pre-existing eye conditions and medical procedures, eye pathology or surgical interventions (laser or classical) in the previous year.

*Ocular Surface Disease Index^©^ (OSDI^©^,* *Allergan Inc., 1995;* *Walt, 2004)* is a 12-item instrument designed for the assessment of symptoms, functional limitations, and environmental factors related to dry eye. This measure includes a grading system that enables the classification of the severity of dry eye disease (normal, mild, moderate or severe). Each item is scored on a Likert-type scale ranging from 0 to 4 points, where 0 indicates ‘none of the time’ and 4 ‘all the time’. Dry eye severity is calculated using the OSDI^©^ formula: OSDI^©^ = (sum of scores) × 25 / (number of questions answered). The calculated OSDI score is expressed on a scale from 0 to 100, where higher scores represent greater disability, expressed as a more frequent and greater number of dry eye symptoms and greater disturbance of normal visual function. Using, the OSDI score, an individual can be categorized as having a normal ocular surface (0–15 points) or mild (16–30 points), moderate (31–44 points) or severe (45–100 points) ocular surface disease. Scores of 31.3 and higher (moderate and severe categories) are considered to be indicative of a dry ocular surface.

For the purposes of this study and in accordance with guidelines for the translation and cultural adaptation of questionnaires (Process of translation and adaptation of instruments, 2010), the OSDI was translated from English to Croatian by optometrists, ophthalmologists and an official English language translator. During the translation process and in preparing the questionnaire for online application, a number of minor changes were made. Namely, response scale descriptors were adapted so that they made more sense in the Croatian language. In addition, response options were presented in the reverse order of that used in the original scale. This was necessary so that the questionnaire was suitable for online application. In the original instrument, the respondent has the option to opt out from answering questions posed in the last two groups of items if he/she does not identify with the described situation. In this case, the total number of items to which the respondent provided an answer is taken into account in the final calculation of the OSDI score. In the present study, participants were required to respond to all 12 items. As such, the number of responses was considered to be constant across all participants when calculating the final OSDI index.

Previous research has established that the OSDI has high internal consistency, excellent test-retest reliability and discriminant validity, good sensitivity and specificity in distinguishing between normal subjects and patients with dry eye disease (Schiffman et al., 2000). In the present sample, the OSDI demonstrated very good internal consistency (*α* = .86).

*Anxiety Sensitivity Index (ASI,* *Peterson & Reiss, 1987)* is a 16-item self-report measure of the fear of sensations associated with arousal. In the current research Croatian version of the measure translated from English (Jurin, Jokic-Begic, & Korajlija, 2012) was used. Each item consists of a possible negative consequence of anxiety symptoms (e.g. It scares me when I feel faint), on which responses are made using a 5-point Likert scale ranging from 0 (very little) to 4 (very much). A higher total score indicates higher levels of anxiety sensitivity. Previous studies have confirmed a hierarchical factor structure of the ASI that consists of three lower-level factors: concerns about physical symptoms of anxiety (physical concerns), concerns about mental capacities (psychological concerns) and social concerns related to fear of public disclosure of anxiety symptoms and shaming (Jurin et al., 2012). In the present sample, the ASI demonstrated very good internal consistency (*α* = .91).

*Depression, Anxiety and Stress Scale (DASS-21;* *Lovibond & Lovibond, 1995)*. Croatian adaptation and translation (Ivezic, Jaksic, Jokic-Begic, & Surányi, 2012) of the English version of the DASS-21 was used to assess depressive and anxious symptoms. For each of the 21 item, participants estimate the degree to which a given statement has applied to them over the past week. All responses are made on a 4-point Likert scale (0 = did not apply to me at all; 3 = applied to me very much). The DASS-21 is composed of three relatively independent subscales measuring levels of depression, anxiety, and stress. The total score and each subscale score additionally represent the measurement of the broader dimension of psychological distress or neuroticism. In the present sample, the DASS demonstrated excellent internal consistency (*α* = .95).

## Bias

The participants were randomly recruited via social networks. Having in mind a possible selection of younger people who are usually dominantly present on social networks in the research, we additionally recruited individuals from older age groups via personal mailing lists with a snowball technique. The participation was anonymous and voluntary. However, this study has some sources of bias which are discussed in the Limitations section.

### Quantitative variables

The Statistical Package for the Social Sciences, version 20.0 (SPSS Inc., Chicago, IL) was used for all data analyses. All data was coded in SPSS 20.0 database. Total composite scores for OSDI, ASI, and its subscales, and DASS-21 and its subscales were calculated. Means and standard deviations for every scale variable were computed.

## Statistical methods

In order to investigate the relationships between DED and socio-demographic and personality variables, a correlation analysis was performed. In order to establish the predictors of DED, hierarchical multiple regression analyses were conducted. The level of statistical significance was set at *p* < .05. The missing data was excluded by the listwise method.

## Results

### Participants

A total of 402 persons responded to the survey. All participants were Caucasian and were living in Croatia at the time of questionnaire completion. Because a number of pre-existing eye conditions and medical procedures can provoke the occurrence of dry eye symptoms occurrence (Ambrósio, Tervo, & Wilson, 2008; De Paiva et al., 2006; Stapleton et al., 2017; Toda, Asano-Kato, Komai-Hori, & Tsubota, 2001), participants who reported experiencing a pathologic eye problem (predominantly physiological, such as conjunctivitis) or surgical interventions (laser or classical) in the previous year were excluded. In addition, because contact lenses can be related to or cause dry eye problems, contact lens wearers were also excluded (Begley et al., 2000; J.J. Nichols & Sinnott, 2006; Stapleton et al., 2017). Following the additional exclusion of any incomplete questionnaires, a total of 381 participants met all criteria for further analysis.

### Descriptive data

256 (67.2%) participants were women. Participant age ranged between 20 and 89 years, with an average age of 39.72 years (*SD* = 12.4). The majority of participants were between 25 and 54 years of age (80.9%), were highly educated (54.3% held a Bachelor's or Master's degree and 13.1% held a doctoral degree) and employed (82.2%). Sample demographics are presented in Table 1.

**Table 1.** Baseline characteristics of the participants (*N* = 381) as a percentage of the sample.

Charachteristic	*n*	*%*
Age		
18–24	19	4.9
25–34	147	38.6
35–44	88	23.1
45–54	73	19.2
55–64	41	10.8
65–74	9	2.4
75 or older	4	1
Gender		
Female	256	67.2
Male	125	32.8
Educational level		
Primary school	3	0.8
High school	121	31.8
BA or MA	207	54.3
PhD	50	13.1
Working status		
Employed	313	82.2
Unemployed	28	7.3
Student	25	6.6
Retired	15	3.9
OSDI category		
Normal (≤15)	162	42.5
Mild (16–30)	132	34.6
Moderate (31–44)	66	17.3
Severe (≥45)	21	5.5

### Outcome data

According to OSDI scores, 57.5% of the sample reported at least a mild level of dry eyes symptoms, and a further 22.8% of participants reported moderate or severe dry eyes symptoms (Table 1). Using the OSDI threshold criterion for the presence of dry eyes (OSDI score: 31.3), 22.8% of participants in this study can be said to have a dry ocular surface. A comparison of the results across genders indicates that 27.3% of women achieve a score higher than 31.3, while this percentage is significantly lower among men (13.6%). Table 2 presents zero-order correlations among the key variables, along with means and standard deviations.

**Table 2.** Correlation matrix of the used variables, means, standard deviations, and ranges (*N* = 381).

Measure	1	2	3	4	5	6	7	8	9	10	*M*	*SD*	Range
1. Gender^a^	–												
2. Age	.14**	–									39.72	12.38	20–89
3. OSDI	−.20**	.12*	–								19.91	14.47	0–72.92
4. ASI	−.17**	−.12**	.33**	–							18.61	11.00	0–60
5. ASI-phy	−.16**	−.01	.27**	.94**	–						9.03	6.54	0–32
6. ASI-psy	−.16**	−.20**	.35**	.89**	.71**	–					5.35	4.35	0–24
7. ASI-soc	−.10	−.20**	.18**	.55**	.37**	.42**	–				4.23	1.82	0–8
8. DASS-21	−.20**	−.10	.28**	.59**	.49**	.60**	.32**	–			10.93	10.61	0–55
9. DASS-dep	−.14**	−.05	.22**	.47**	.36**	.49**	.29**	.91**	–		3.47	4.09	0–21
10. DASS-anx	−.09	−.06	.28**	.58**	.51**	.56**	.28**	.87**	.68**	–	2.29	3.12	0–18
11. DASS-stress	−.27**	−.13*	.31**	.58**	.49**	.59**	.30**	.93**	.76**	.74**	5.2	4.45	0–20

Note*:* ^a^female = 1, male = 2; OSDI – Ocular Suface Disease Index; ASI – Anxiety Sensitivity Index, ASI-phy – ASI Physical Concerns subscale, ASI-psy – ASI Psychological Concerns subscale, ASI-soc – ASI Social Concerns subscale; DASS-21 – Depression, Anxiety, and Stress Scale; short form, DASS-dep – DASS-21 depression subscale; DASS-anx – DASS-21 anxiety subscale; DASS-stress – DASS-21 stress subscale; *p < .05, **p < .01.

### Main results

All variables are significantly correlated with OSDI scores. Symptoms of dry eyes are more pronounced among women and older participants. Furthermore, ASI scores were significantly and positively correlated with OSDI scores (*r* = .33, *p* < .01). When the three ASI dimensions are examined, ASI-psychological concerns is most highly correlated with OSDI scores (*r* = .35, *p* < .01), followed by ASI-physical concerns (*r* = .27, *p* < .01), and ASI-social concerns (*r* = .18, *p* < .01).

Significant relationships were also found between OSDI scores and DASS-21 scores, as well as between OSDI scores and two factors of the DASS-21 (depressiveness and stress).

Correlational analysis was followed by hierarchical multiple regression analyses. In order to assess the robustness of the association between anxiety sensitivity and DED symptoms, ASI scores were entered into a hierarchical linear regression equation with OSDI as the dependent variable and gender, age and DASS-21 score as covariates. This allowed for the determination of whether general depressive and anxiety symptoms might account for the link between AS and symptoms of DED. The results of this analysis revealed that, after controlling for gender, age and DASS-21 total scores, the association between OSDI scores and ASI total scores was still significant (*β* = .23, *t*(329) = 3.64, *p* < .01). Results of this hierarchical regression analysis are presented in Table 3.

**Table 3.** Hierarchical multiple regression analyses predicting OSDI Score from demographic variables, DASS and ASI scores.

Predictor	Δ*R*²	*β*	95% CI
Step 1	.07**		
Gender		−.23**	[−.34, −.13]
Age		.14*	[.03, .25]
Step 2	.07**		
Gender		−.18**	[−.29, −.08]
Age		.16**	[.06, .27]
DASS-21		.26**	[.16, .37]
Step 3	.03*		
Gender		−.17**	[−.28, −.07]
Age		.17**	[.08, .28]
DASS-21		.13*	[.01, .26]
ASI		.23**	[.11, .36]
Total *R*²	.17**		
*N*	334		

Note: OSDI – Ocular Surface Disease Index; DASS-21 - Depression, Anxiety, and Stress Scale, short form; ASI – Anxiety Sensitivity Index; *p *<* 0.05, **p *<* 0.01.

Within a more exploratory framework, we also aimed to examine the predictive patterns of psychological factors, and the subcomponents of AS in particular, for the occurrence of DED. Previous research has found that ASI factors often demonstrate differing sensitivity in predicting various symptoms and behavioral responses (Brown, Smits, Powers, & Telch, 2003). The data presented in Table 2 indicates that the subscales of the DASS-21 and the ASI were significantly correlated with OSDI scores (*p*s < .01 for all correlations). In the final phase of analysis, we again constructed a regression equation with gender, age and DASS-21 subscales as covariates and found that the relationship between the psychological concerns dimension of the ASI and OSDI scores remained significant (*β* = .30, *t*(325) = 3.82, *p* < .01). Specifically, symptoms of DED were found to be more intense among women and older participants who scored higher on the psychological dimension of anxiety sensitivity. The results of this analysis are presented in Table 4.

**Table 4.** Hierarchical multiple regression analyses predicting OSDI score from demographic variables, DASS and ASI scores.

Predictor	Δ*R*²	*β*	95% CI
Step 1	.07**		
Gender		−.23**	[−.34, −.13]
Age		.14*	[.03, .25]
Step 2	.08**		
Gender		−.18**	[−.29, −.07]
Age		.17**	[.07, .28]
DASS-dep		−.04	[−.20, .12]
DASS-anx		.15	[−.01, .30]
DASS-stress		.19*	[.01, .38]
Step 3	.06**		
Gender		−.17**	[−.28, −.07]
Age		.22**	[.13, .34]
DASS-dep		−.09	[−.24, .07]
DASS-anx		.08	[−.08, .24]
DASS-stress		.12	[−.07, .30]
ASI-phy		−.07	[−.22, .07]
ASI-psy		.30**	[.15, .46]
ASI-soc		.08	[−.02, .19]
Total *R*²	.20**		
*N*	334		

Note: OSDI – Ocular Surface Disease Index, DASS-21 – Depression, Anxiety, and Stress Scale; short form, DASS-dep – DASS-21 depression subscale; DASS-anx – DASS-21 anxiety subscale; DASS-stress – DASS-21 stress subscale, ASI – Anxiety Sensitivity Index, ASI-phy – ASI Physical Concerns subscale, ASI-psy – ASI Psychological Concerns subscale, ASI-soc- ASI Social Concerns subscale, *p < .05, **p < .01.

## Discussion

### Key results

The present study recorded a prevalence of 22.8% of moderate or severe dry eye symptoms based on a convenient sample of the general adult population in Croatia.

To our knowledge, this is the first study to examine AS as a possible psychological trait risk factor for DED symptoms. This study provides at least partial support for the hypotheses that AS may serve as an important vulnerability factor for DED symptoms. The findings demonstrated that AS, and AS-psychological concerns, in particular, predict the intensity of dry eye symptoms above and beyond depressive and anxiety symptoms.

### Limitations

There are a number of limitations to the present study that should be taken into consideration. First, because any measure used for the diagnosis of DED must include objective signs of dry eyes, the survey used in this research was not designed to be used for diagnostic purposes. Because of the strict exclusion criteria used in the study, persons who reported any external reasons for dry eye symptoms (e.g. contact lens use, inflammation, surgery or similar) were excluded. In this way, the most severe cases, such as those in which the onset of ocular surface inflammation was a result of eye dryness, were perhaps excluded. Second, the sample used in the present study represented a convenience sample recruited from the general community. This study also holds all limitations related to the use of an online data collection method, such as the self-selection of participants, which undermines the external validity of the study and the interpretation of the findings (Fenner et al., 2012). Previous research has concluded that such biases have the potential to distort point-estimates, such as average symptom level or prevalence, but not patterns of associations with putative risk-factors (Heiervang & Goodman, 2011). Nevertheless, repeating the study with a clinical sample of ophthalmological, depressive and anxious patients would be of great importance. It should also be noted that the sample was predominantly female and middle-aged. Because female gender is a risk factor for DED, this over-representation of women is, in some way, representative of the general population of DED patients. However, further research should be carried out using samples more representative of the general population and especially samples with a greater representation of older individuals, who have been shown to have greater prevalence of dry eye problems. Third, the cross-sectional nature of the study precludes any causal inferences. Previous research has suggested that AS arises as a result of biological vulnerabilities and learned beliefs about the catastrophic consequences of interoceptive sensations (Dixon et al., 2016). Prospective, longitudinal studies are necessary to examine the exact nature of the relationship between AS and DED. In addition, the use of newer measures of AS, such as the ASI-3 (Taylor et al., 2007) in future research examining this relationship might also be helpful. Finally, the single-method self-report research design also raises the possibility that a shared method of variance might have partially contributed to the observed findings.

### Interpretation

Dry eye disease is a common health problem in the general population. Previous studies have demonstrated that the subjective symptoms of dry eye are associated with several psychological factors, including depression, anxiety, post-traumatic stress disorder and subjective feelings of happiness (Ichinohe et al., 2016; Jonas et al., 2018; Kawashima et al., 2015; Kim et al., 2011; Mizuno et al., 2010; Shtein et al., 2016; Stapleton et al., 2017; Um et al., 2018; Wan et al., 2016; Wen et al., 2012; Yilmaz et al., 2015).

In the present study, 22.8% of the participants reported a dry ocular surface. As expected, women more often reported symptoms of dry eye that could be categorized as moderate to severe. While the results from the present study cannot be fully compared to previous research because of differences across studies in dry eye criteria, diagnostic procedures or participant population our findings are consistent with expected OSDI-based results related to the prevalence of dry eyes. In an American study of DED using a self-report questionnaire, Paulsen et al. (2014) reported a prevalence of 14.5%. Two Japanese studies assessing DED using a questionnaire reported prevalence rates ranging between 15.3% and 17% (Toda, Fujishima, & Tsubota, 1993) and 33% (Shimmura et al., 1999), respectively. The results in the present study represent the approximate mid-point in this reported range.

Our findings demonstrated that AS, and especially AS-psychological concerns, predict the intensity of dry eye symptoms above and beyond depressive and anxiety symptoms. This is consistent with the previous results demonstrating that heightened AS may serve to increase the chance that an individual will detect, experience and report physical symptoms (Smitherman et al., 2015). Apart from any direct correspondence between the evaluation of somatic events and objective health status, attention to and reporting of symptoms is important in that these behaviors represent key components of health maintenance and decisions related to seeking medical treatment, as well as general health-protective behaviors.

It appears interesting that the ASI-physical concerns dimension is not as strongly associated with the OSDI as the ASI-psychological concerns dimension. One might expect so because OSDI as the outcome measure captured physical symptoms of DED. However, the results are somewhat logical, and there are a few possible explanations for it.

First, ASI-physical concerns dimension does not capture any of eye-related symptoms like OSDI, but few other body sensations like palpitations and stomachache. Eye-related symptoms are quite specific and mostly marginalized or ignored in the instruments examining somatization symptoms.

Second, most of the research confirm a lack of or weak correlation of physical signs of dry eye and perceiving symptoms of dry eye (Baudouin et al., 2014; Begley et al., 2003; Galor et al., 2015; Kawashima et al., 2015; Lavrič & Olup, 2010; Mizuno et al., 2010; K.K. Nichols et al., 2004; Pult et al., 2011; Schein et al., 1997; Schiffman et al., 2000) and other reports often significant associations with psychological conditions (Galor et al., 2015; Hyon et al., 2019; Ichinohe et al., 2016; Jonas et al., 2018; Kawashima et al., 2015; Kim et al., 2011; Mizuno et al., 2010; Stapleton et al., 2017; Um et al., 2018; Wan et al., 2016; Wen et al., 2012; Yilmaz et al., 2015) which indicates that there may be a special mechanism underlying this symptom different from the other body sensations and more with psychological symptoms as described in the items of ASI-psychological concerns.

In a recently published systematic review examining the pathways of AS and medical conditions, it is postulated that AS increases the risk for chronic medical conditions via fear amplification and shared vulnerability (Horenstein et al., 2018). In the case of DED, a heightened focus on symptoms such as eye fatigue, red eyes, blurred vision and feeling that there is a foreign body in the eye might provoke a sense of concern for mental health because of the common occurrence of these symptoms in states of sadness and depression. Previous research has indicated the presence of subjective visual impairment among depressed patients (Bubl, Tebartz Van Elst, Gondan, Ebert, & Greenlee, 2009; Friberg & Borrero, 2000) as well as a relationship between lower functionality and well-being and blurred vision (Lee, Spritzer, & Hays, 1997). It might be argued that people with heightened anxiety sensitivity are more likely to perceive symptoms of dry eyes and subsequently associate these symptoms with what they have learned from experience, that is, that such symptoms arise during periods of depressed mood, fatigue, and general distress. When such interpretations additionally focus attention on the symptoms of the eyes and eye surrounding areas, the individual is also likely to seek the help of a physician. Negative cognitive appraisal plays a role in the establishment and maintenance of anxiety and stress which, in turn, exacerbate psychosomatic symptoms including symptoms of DED. This is perhaps is one plausible explanation for the widely reported discordance between the symptoms and signs of DED (see Baudouin et al., 2014; Begley et al., 2003; Galor et al., 2015; Kawashima et al., 2015; Lavrič & Olup, 2010; Mizuno et al., 2010; K.K. Nichols et al., 2004; Pult et al., 2011; Schein et al., 1997; Schiffman et al., 2000) and the larger association between DED symptoms and psychological difficulties (see Galor et al., 2015; Hyon et al., 2019; Ichinohe et al., 2016; Jonas et al., 2018; Kawashima et al., 2015; Kim et al., 2011; Mizuno et al., 2010; Stapleton et al., 2017; Um et al., 2018; Wan et al., 2016; Wen et al., 2012; Yilmaz et al., 2015) than with actual signs of DED. Similarly, such individuals might be more concerned for their own mental health because symptoms related to their eyes and vision are interpreted as signs of mental difficulty, which in turn directs further attention to one’s own mental state. Arguably, this might explain why patients with DED who report symptoms of dry eye exhibit greater levels of anxiety and depression than DED patients who have signs of the disease, but no symptoms. (Vehof et al., 2016). A recent study (Asiedu et al., 2018) demonstrated that the severity of dry eye symptoms, but not signs, had an impact on psychosomatic symptoms and quality of life. This study also indicated that the severity of dry eye symptoms had a greater impact on depressive symptoms than other psychosomatic symptoms. Similarly, a large clinical study demonstrated that discordance between the symptoms and signs of DED is an indicator of self-perceived health (Vehof et al., 2016). This study demonstrated that the presence of a chronic pain syndrome, atopic disease, depression, and antidepressant use were significant predictors of a sign-symptom dissonance in which symptoms were greater than the signs of DED. Predictors of the inverse relationship (i.e. fewer symptoms than signs) were increased age and the presence of Sjögren's syndrome. The results of the present study are consistent with the contemporary hypothesis that a central pathophysiologic mechanism contributes to eye pain symptoms in this population (Shtein et al., 2016).

Is it possible to conclude that AS is a vulnerability factor for DED? In order to declare a given variable a risk factor, three criteria must be met (Garber & Hollon, 1991). First, the proposed vulnerability factor must be correlated with the construct of interest – in this case, symptoms of DED. Second, the relationship between the proposed vulnerability factor and DED should not be better explained by a third factor. Third, the vulnerability factor should demonstrate temporal precedence. The present study aimed to investigate whether AS satisfies the first two criteria, thereby providing partial evidence that these factors may serve as risk factors for the symptoms of DED.

Despite the limitations, the findings in this study expand current understanding of psychological mechanisms contributing to dry eye disease. Previous studies have indicated that AS is associated with increased fear of medical condition-specific symptoms, the avoidance of and interference with behaviors that promote physical well-being and engagement in behaviors that are detrimental to physical health (Horenstein et al., 2018). This is the first study to suggest that AS, and AS-psychological concerns in particular, is incrementally related to dry eye symptoms above and beyond other relevant variables (e.g. anxiety, depression and stress). However, longitudinal studies are needed to clarify the temporal sequencing of AS and DED. Further establishing the relationship between AS and objective measures of DED should also be an area of focus for future research. A recent study (Shtein et al., 2016) revealed a subgroup of patients with DED exhibiting a unique disease mechanism that extends beyond the ocular surface–lacrimal gland complex. This discordant subgroup (defined as those with dry eye symptoms but a normal result on an objective test of ocular surface) exhibited decreased corneal nerve density and decreased visual quality-of-life scores, features also observed among patients with fibromyalgia. The authors propose that, among individuals with discordant DED, central neural processes are responsible for much of the discordance between symptom severity and the degree of ocular surface damage (Shtein et al., 2016). It might be argued that these basic mechanisms are responsible for increased interoceptive consciousness and anxiety sensitivity.

Although our understanding of the relationship between AS and DED is only in an early phase, a number of practical recommendations for assessment and treatment can be given based on the findings of the present study. First, administering an AS questionnaire (the ASI-3 is recommended) with DED patients might help identify those at risk for the development of more prominent symptoms and comorbid conditions of depression and anxiety. Because persons with symptoms of DED will most often seek help from a general practitioner or ophthalmologist and do not typically seek specialty mental health services, it might be especially useful to incorporate assessments of AS into primary care visits. Fortunately, a number of empirically supported treatments such as psychoeducation, cognitive restructuring and interoceptive exposure have been demonstrated to be effective in reducing anxiety sensitivity (Watt, Stewart, Lefaivre, & Uman, 2006).

### Generalisability

Although we invested an additional effort to achieve a greater level of sample heterogeneity this study has limitations related to the use of an online data collection method. Considering that and the fact that participants were conveniently sampled for the study findings cannot be generalized to the entire population of adults in Croatia.
